# First Step on the Way to Identify Dermatophytes Using Odour Fingerprints

**DOI:** 10.1007/s11046-024-00905-7

**Published:** 2025-01-07

**Authors:** Lenka Machová, Meriem Gaida, Jaroslav Semerád, Miroslav Kolařík, Michaela Švarcová, Andrej Jašica, Alena Grasserová, Sandra Awokunle Hollá, Vit Hubka, Pierre-Hugues Stefanuto, Tomáš Cajthaml, Jean-François Focant, Adéla Wennrich

**Affiliations:** 1 Laboratory of Fungal Genetics and Metabolism, Institute of Microbiology of the Czech Academy of Sciences, Prague, Czech Republic; 2 Department of Genetics and Microbiology, Faculty of Science, Charles University, Prague, Czech Republic; 3 Organic and Biological Analytical Chemistry Group, Chemistry Department, University of Liège, Liège, Belgium; 4 Laboratory of Environmental Biotechnology, Institute of Microbiology of the Czech Academy of Sciences, Prague, Czech Republic; 5 Institute for Environmental Studies, Faculty of Science, Charles University, Benátská 2, Prague 2, 12801 Czech Republic; 6 Department of Botany, Faculty of Science, Charles University, Prague, Czech Republic; 7 Laboratory of Environmental Microbiology, Institute of Microbiology of the Czech Academy of Sciences, Prague, Czech Republic

**Keywords:** Dermatophytes, Volatile organic compounds, Metabolite profiles, Gas chromatography-mass spectrometry

## Abstract

The clinical diagnosis of dermatophytosis and identification of dermatophytes face challenges due to reliance on culture-based methods. Rapid, cost-effective detection techniques for volatile organic compounds (VOCs) have been developed for other microorganisms, but their application to dermatophytes is limited. This study explores using VOCs as diagnostic markers for dermatophytes. We compared VOC profiles across different dermatophyte taxa using solid-phase microextraction (SPME) and advanced analytical methods: gas chromatography-mass spectrometry (GC–MS) and comprehensive two-dimensional gas chromatography with time-of-flight mass spectrometry (GC×GC-TOFMS). We analyzed 47 dermatophyte strains from 15 taxa grown on sheep wool, including clinically significant species. Additionally, we examined phylogenetic relationships among the strains to correlate genetic relatedness with metabolite production. Our results showed that GC×GC-TOFMS offered superior resolution but similar differentiation of VOC profiles compared to GC–MS. VOC spectra allowed reliable distinction of taxonomic units at the species level and below, however, these distinctions showed only a slight correlation with phylogenetic data. We identified pan-dermatophyte and species- or strain-specific VOC profiles, indicating their potential for rapid, non-invasive detection of dermatophyte infections, including epidemic strains. These patterns could enable future taxa-specific identification. Our study highlights the potential of VOCs as tools for dermatophyte taxonomy and diagnosis.

**Supplementary Information:**

The online version contains supplementary material available at 10.1007/s11046-024-00905-7.

## Introduction

Dermatophytes are the most common agents that cause fungal skin infections in mammals [1, 2]. Identification of the dermatophyte species, hosts and reservoirs is very important during local epidemics. As drug-resistant populations continue to emerge and the possibility of new pathogens being introduced increases, there is increasing pressure on clinical mycologists to use state-of-art diagnostic tools [3, 4]. The majority of dermatologists use a combination of clinical assessment, cultivation and direct microscopy for treatment assessment [5]. The most used technique for identifying dermatophyte species is based on phenotypic criteria by microscopic examination [6]. However, this method requires a high level of expertise from clinical workers, is time-consuming, and often leads to misdiagnosis due to the unstable nature of the phenotype [7]. There are alternative methods, such as matrix-assisted laser desorption/ionization with time-of-flight mass spectrometry (MALDI-TOFMS) and molecular-based detection methods, that are gradually replacing phenotypic identification in clinical practice [8–11]. These methods are generally effective, but they usually require a problematic cultivation step, with a few exceptions. Polymerase chain reaction (PCR)-based methods are currently the most successful and developed tools for directly identifying dermatophytes from tissues. These methods, such as real-time PCR assays or probe hybridizations, have been successfully used to identify dermatophytes from paraffin sections, skin scrapings, nail, and hair samples in several cases [9, 12–14]. However, these identification assays are only designed for a limited range of dermatophyte species and a few non-dermatophyte pathogens. They also do not capture variability at the population level, making it difficult to identify and monitor new clinical entities. These methods are not widely used due to the complex sample processing required, often in specialized laboratories. Recently, techniques using the detection of volatile organic compounds (VOCs) have been developed to address the need for non-invasive and rapid diagnostics. VOCs are solid and liquid substances that quickly turn into gas at low pressure and normal laboratory temperature [15]. These properties allow VOCs to easily spread into the surrounding environment of growing microorganisms, making them detectable. VOC research initially began in environmental studies but gained attention in the 1990s for its potential in diagnosing metabolic diseases like diabetes and cancers. By the 2000s, VOCs were being explored for identifying pathogens due to their ability to produce distinct metabolic profiles. Today, VOC detection offers a non-invasive alternative to biopsies or culturing, with the potential for real-time diagnostics in clinical settings [16]. This approach is also being tested for diagnosing metabolic diseases and cancers, where altered metabolic pathways produce unique VOC profiles [17, 18]. In dermatology, it shows promise for identifying bacterial and fungal infections, though further research is needed to improve its sensitivity and specificity [19]. This method could significantly reduce diagnostic times and improve treatment accuracy across various medical conditions. Additionally, such a solution does not impose significant financial, or material demands on medical institutions [20]. Currently, the detection of these substances requires the use of chromatography methods coupled with mass spectrometry. However, small portable devices known as "electronic noses" could be an alternative to these time and space-consuming methods [16]. This technique can selectively identify specific markers. For instance, devices used for quality checks in the food industry demonstrate that there is no need for sophisticated machinery to detect specific VOCs [21]. Consequently, electronic noses, which are based on the detection of specific molecules produced by microorganisms, could potentially become a standard routine in clinical practice. This suggests a promising future for simple yet effective detection methods. VOCs as well as other metabolites are frequently species-specific in fungi. Before the molecular era, chemotaxonomy has traditionally been used as an additional tool for species identification, for example, in lichens, and in the food industry to identify mycotoxin producers [22–26]. VOC markers that can be used in pathogen detection have already been published for bacterial infections [27], aspergillosis [28, 29], candidiasis [30], and other diseases [31]. However, such potential diagnostic markers have not been discovered for dermatophytosis, although electronic noses have already been tested as a fingerprint method on a limited dataset of three to four dermatophyte species and showed possible taxa specificity [32, 33]. To identify species-specific VOCs, gas chromatography coupled with mass spectrometry (GC–MS) is commonly used [34]. However, in recent years, comprehensive two-dimensional gas chromatography coupled with time-of-flight mass spectrometry (GC×GC-TOFMS) has become popular for environmental forensics and metabolomic analysis of microorganisms [35, 36]. The main advantage of GC×GC over one-dimensional GC is its ability to achieve higher resolution. However, this technique requires more complex data processing often involving machine learning algorithms, to extract the most meaningful chemical information [37, 38].

In this study, we used large datasets of dermatophytes. We used two approaches to analyse the spectra of VOCs: gas chromatography–mass spectrometry and two-dimensional gas chromatography with time-of-flight mass spectrometry. Our goal was to examine the specificity of the compounds produced as markers for both pan-dermatophyte diagnostics and diagnostics of individual species.

## Materials and Methods

### Strain and Culture Conditions

A total of 47 strains belonging to 15 taxa of dermatophytes were obtained from both human and animal dermatophytosis cases as well as from culture collections (Table 1). For comparison, three non-dermatophyte fungal species that are frequently found on human skin as contaminants or pathogens were selected: *Aspergillus fumigatus* CCF 3522, *Paecilomyces variotii* CCF 3230 and *Scopulariopsis brevicaulis* CCF 6436. All strains were maintained on Malt Extract Agar (MEA; Sigma-Aldrich, St. Louis, USA) at 6 °C throughout the study.

**Table 1:** List of strains examined in this study

Species	Strain designations^1^	Year of isolation/origin	Analysis type	GenBank/ENA/DDBJ accession numbers		
				*ITS*	*tubb*	*tef1a*
*Trichophyton benhamiae* var. *benhamiae*	IHEM 4710	Unknown/USA	GC–MS	LR794129	LR794285	LR794260
	CCF 6486	1995/USA	GC–MS	LR794130	LR794286	LR794261
	IHEM 3287	1970/Belgium	GC–MS	LR794129	–	LR794260
*Trichophyton benhamiae* var.* luteum*	CLIS 6433/20	2020/Czechia	GC–MS; GCxGC-TOFMS	OR619675	OR792381	OR825132
	CLIS 9200/20	2020/Czechia	GC–MS; GCxGC-TOFMS	OR619678	OR792383	OR825134
	CLIS 9106/20	2020/Czechia	GC–MS; GCxGC-TOFMS	OR619677	OR792382	OR825133
	SK 2597/20	2020/Czechia	GCxGC-TOFMS	LR794131	–	–
	CLIS 8587/19	2019/Czechia	GCxGC-TOFMS	LR794131	–	–
*Trichophyton europaeum*	CLIS 5085/20	2020/Czechia	GC–MS	OR619670	OR792376	OR825119
	CLIS 4898/20	2020/Czechia	GC–MS	OR619668	OR792374	OR825117
	SK 586/20	2020/Czechia	GC–MS	OR619693	–	–
*Trichophyton japonicum*	CLIS 9483/18	2018/Czechia	GC–MS	OR619679	–	–
	L 1430/20	2020/Czechia	GC–MS	OR619684	OR792386	OR825128
	SK 3908/19	2019/Czechia	GC–MS	OR619692	OR792388	OR825130
*Trichophyton persicum*	CCF 6543	2017/Iran	GC–MS	MG356864	MG356864	MW959139
*Trichophyton erinacei*	CCF 6504	2019/Czechia	GC–MS; GCxGC-TOFMS	OR619666	OR792373	OR825116
	CCF 6399	2019/Czechia	GC–MS; GCxGC-TOFMS	OR619665	OR792372	OR825115
	CLIS 3198/20	2020/Czechia	GC–MS; GCxGC-TOFMS	MZ314454	MZ320337	MZ320327
	CCF 4472	2012/Czechia	GCxGC-TOFMS	LR794136	–	–
	CCF 6563	2017/Japan	GCxGC-TOFMS	OR619664	–	OR825114
*Trichophyton verrucosum*	L 28/19	2019/Czechia	GC–MS	OR619685	OR792387	OR825129
	CLIS 7396/20	2020/Czechia	GC–MS	OR619676	–	–
*Trichophyton quinckeanum*	CLIS 9771/20	2020/Czechia	GC–MS	OR619680	OR792384	OR825123
	CLIS 6248/20	2020/Czechia	GC–MS	OR619674	OR792380	OR825122
*Microsporum canis*	D 106/20	2020/Czechia	GC–MS	OR619681	OR792390	OR825135
	CLIS 2072/20	2020/Czechia	GC–MS	OR619667	–	–
*Trichophyton mentagrophytes*	CLIS 6191/20	2020/Czechia	GC–MS	OR619673	OR792379	OM568761
	PL 700/20	2020/Czechia	GC–MS	OR619689	OM314973	OM568761
	CCF 6572	2017/Czechia	GC–MS; GCxGC-TOFMS	OM283516	OM568760	OM314972
	CCF 6579	2017/Czechia	GC–MS; GCxGC-TOFMS	OM283522	OM568766	OM314978
	CCF 6573	2016/Czechia	GCxGC-TOFMS	OM283517	OM314973	OM568761
	CCF 6574	2016/Czechia	GCxGC-TOFMS	OM283518	OM314974	OM568762
	CCF 6584	2015/Czechia	GCxGC-TOFMS	OM283528	OM314984	OM568776
*Trichophyton mentagrophytes* var. *indotineae*	CCF 6599	2016/Czechia	GC–MS	OM283512	OM568780	OM314968
	CCF 6597	2019/Japan	GC–MS	OM283543	OM568778	OM314999
	CCF 6598	2019/Japan	GC–MS	OM283544	OM568779	OM315000
*Trichophyton rubrum*	LY 8	before 2016/Denmark	GC–MS	OR619687	–	–
	LY 19	before 2016/Denmark	GC–MS	OR619686	–	–
	SK 701/20	2020/Czechia	GC–MS	OR619694	OR792389	OR825131
	IDE 241/20	2020/Czechia	GC–MS	OR619683	OR792385	OR825127
*Trichophyton tonsurans*	CLIS 5203/20	2020/Czechia	GC–MS	OR619671	OR792377	OR825120
	CLIS 5011/20	2020/Czechia	GC–MS	OR619669	OR792375	OR825118
	CLIS 5356/20	2020/Czechia	GC–MS	OR619672	OR792378	OR825121
*Epidermophyton floccosum*	PL 782/20	2020/Czechia	GC–MS	OR619688	OR792392	–
*Nannizzia gypsea*	SK 2458/20	2020/Czechia	GC–MS	OR619691	OR792394	OR825126
	SK 2316/20	2020/Czechia	GC–MS	OR619690	OR792393	OR825125
	D 653/20	2020/Czechia	GC–MS	OR619682	OR792391	OR825124

^1^Acronyms used for culture collection/clinician providing the strains: IHEM: *Belgian Coordinated Collections of Micro-organisms, Fungi Collection: Human and Animal Health, Sciensano, Brussels, Belgium; CCF: Culture Collection of Fungi, Department of Botany, Charles University, Prague, Czech Republic; CLIS: Public Health Institute in Ostrava, Ostrava, Czech Republic; SK: Department of Dermatology and Venereology, First Faculty of Medicine, General University Hospital in Prague, Charles University and General University Hospital in Prague, Prague, Czech Republic; LY/PL: Public Health Institute in Ústí Nad Labem, Prague, Czech Republic; L: Regional Hospital Liberec; D: Laboratory of Medical Parasitology and Mycology, Hospital České Budějovice, České Budějovice, Czech Republic; RK: Teikyo University Institute of Medical Mycology (TIMM), Tokyo, Japan; IR-ARM: Cellular and Molecular Research Center, Medical Basic Sciences Research Institute, Ahvaz Jundishapur University of Medical Sciences, Ahvaz, Iran; IDE: Department of Microbiology, Palacký University in Olomouc, Faculty of Medicine; USA: University of Illinois at Urbana-Champaign, USA*

### Cultivation and Pre-treatment: GC–MS Analysis

The strains were pre-cultivated in 500 ml Florence flasks with 100 ml of Czapek-Dox broth (Sigma-Aldrich, St. Louis, USA), 0.1 g of unprocessed autoclaved sheep wool, 0.088 g/l of thiamine hydrochloride (Sigma-Aldrich, St. Louis, USA) and 0.096 g/l of glycine (Sigma-Aldrich, St. Louis, USA). The flasks were shaken at 200 rpm and incubated at 37 °C for 10 days. Control samples were prepared identically, but they were not inoculated. After vigorous shaking, 0.5 ml of the pre-cultivation media was inoculated into 20 ml glass vials. These vials contained a PTFE/silicone septum and an autoclaved mixture of 0.1 g of unprocessed sheep wool with 2 ml of 2% agar (Sigma-Aldrich, St. Louis, USA) for firming properties. The vials were then cultivated at 30 °C for 30 days. This length of cultivation time was chosen because it provided better detectability of VOCs in the headspace compared to the two-week and three-week cultivations performed in the initial pilot study (data not shown). Three biological replicates per each strain were studied.

### Cultivation and Pre-treatment: GC×GC-TOFMS Analysis

Compared to GC–MS analysis, the dataset was reduced to focus on a more detailed analysis of three specific taxa due to computational requirements. The zoophilic species *T. erinacei*, *T. mentagrophytes* var. *mentagrophytes*, and *T. benhamiae* var*. luteum* were chosen based on their significance in veterinary medicine. These species were cultivated on 60 mm plates using a 1.5% MEA medium for one week. Afterwards, 2 ml of 0.1% Tween 80 (Lach-Ner, Neratovice, Czech Republic) PBS (Phosphate Buffer Saline; Sigma-Aldrich, St. Louis, USA) was added to the plates. The plates were then swabbed with a sterile cotton swab and resuspended in 6 ml of sterile 0.1% Tween 80 PBS. Each suspension was vortexed and diluted to approximately the same density. The suspension was then left at room temperature for 5 min to allow the heavier hyphal elements to settle. From the upper part of the suspension, 1 ml was taken and added to 100 ml of Czapek-Dox broth medium with 0.1 g of unprocessed autoclaved sheep wool, 0.088 g/l of thiamine hydrochloride (Sigma-Aldrich, St. Louis, USA), and 0.096 g/l of glycine (Sigma-Aldrich, St. Louis, USA). The cultivation was performed in triplicate. Control samples were prepared using the same media without the inoculum and processed in the same manner. The strains were cultivated at 37 °C with shaking at 200 rpm. The presence of glucose in the medium was measured daily using a glucose strip test (DIAPHAN®, Erba Lachema, Czech R.). The cultures were filtered two days after glucose depletion. The length of the cultivation period varied from 10 to 13 days for *T. benhamiae* var. *luteum*, 7 to 15 days for *T. erinacei*, and 7 to 9 days for *T. mentagrophytes*. Once the fungi had depleted the glucose, they were expected to primarily digest keratin and produce the highest number of secondary metabolites. Filtration was done using a double layer of sterile Miracloth filtration material (Merck, Rahway, USA) to remove large mycelium fragments. The remaining conidia and small hyphal elements were then removed using the Merck-Millex 0.22 µm syringe filter (Merck, Rahway, USA). The sterile filtrate was frozen at −35 °C until analysis. The defrosted filtrate from the cultivations was pipetted into 4 ml portions in 20 ml headspace vials. A quality control sample was prepared by combining 1 ml from each sample, and the mixture was aliquoted into headspace vials with 5 ml in each and frozen. The quality control sample was used in each measurement to monitor any deviations in the analysis process.

### GC–MS Analysis

Sample analysis was performed using a gas chromatograph 450-GC and ion trap mass spectrometer 240-MS (GC–MS; both Varian, Walnut Creek, CA, USA). Prior to the analysis, the HS-SPME (Headspace-Solid Phase Microextraction) technique was performed using a CombiPal autosampler (CTC Analytics AG, MN, USA) equipped with a heating station, SPME fiber holder, and a 65 µm PDMS/DVB SPME fiber (Supelco, Bellefonte, PA, USA). The measuring conditions for the GC–MS analysis, extraction, and desorption steps were adopted from Semerád et al. [39] with modifications. A vial containing a strain cultivated for 30 days on sheep wool was transferred to the heating station and conditioned for 30 min at 40 °C. Then, the SPME fiber was inserted into the headspace of the vial for 30 min at 40 °C to extract the analytes. The injector and SPME liner (Topaz, Restek, Bellefonte, PA, USA) temperature was set to 250 °C to desorb the analytes from the SPME fiber. After each injection, the SPME fiber was left in the injector for 10 min at 250 °C to prevent sample cross-contamination. The separation of selected analytes was performed on a DB-5MS column (30 m × 0.25 mm id, 0.25 μm df; Agilent Technologies, Santa Clara, CA, USA). Helium was used as the carrier gas for GC in a constant flow of 1.0 ml/min. The temperature gradient was as follows: 40 °C (2 min isothermal), 200 °C (10 °C/min), 260 °C (25 °C/min, 5 min isothermal). The total time for one GC–MS analysis was 25 min and 24 s. The mass spectrometer temperatures for the ion trap, manifold, and transfer line were set to 240, 50, and 280 °C, respectively. The collected data ranged from 50 to 500 m/z. The software used for data acquisition was MS Workstation 6.9.1 (Agilent Technologies, Santa Clara, USA).

### Data Analysis (GC–MS Analysis)

Data were processed using software AMDIS v.2.66 (NIST, Gaithersburg, USA) and Mass Profiler Professional v. 15.1 (Agilent Technologies, Santa Clara, CA, USA), and OpenChrom v. 1.5.0 [40]. The Mass Profiler Professional software generated a table containing 387 variables. Redundant peaks, which were contamination from the column, SPME fiber, and medium, were removed from the data by comparing the sample data with controls. Data were manually checked and corrected by comparison with chromatograms and results of AMDIS analysis. Peak areas were log10 transformed and normalized by probabilistic quotient normalization (PQN) [41]. Compound identification was conducted by comparing the obtained spectra against the NIST20 library (RRID:SCR_006452), similarity values greater than 750 were tentatively identified, and values below 750 were considered unknowns (Unk). Data from the GC–MS analysis are provided in Supplementary files 2 and 4. Principal Component Analysis (PCA) and Hierarchical Clustering Analysis (HCA) were applied to normalized data to analyze the volatile organic compound spectra. HCA used Ward’s method with Euclidean distances to evaluate clustering patterns, while PCA was conducted to compare GC–MS and GC×GC-TOFMS analysis results. The Mantel statistic was used to assess the correlation between GC–MS data and phylogenetic analysis. Only the maximum value from each triplicate of GC–MS analysis was included. A new phylogenetic tree, from which phylogenetic relatedness was calculated, was generated using only taxa that were also present in the GC–MS analysis, enabling comparison between datasets. To qualitatively inspect the GC–MS data, the results were converted to categorical data by classifying compounds in the dermatophyte headspace as either present or absent. The data were divided into three categories based on abundance level (present in all, present in some replicates, not present). All statistical analysis and data visualization were performed using R v. 4.2.1 [42]. For the statistical analysis, the packages stats, reshape2 [43], pvclust [44], FactoMineR [45], ape [46], ggtree [47], vegan [48], and factoextra [49] were utilized. For data visualization, the packages from Tidyverse [50], especially ggplot2 [51], viridis [52], gridExtra [53], and cowplot [54] were employed.

### HS-SPME-GC×GC-TOFMS Analysis

All extractions were performed using the triphasic DVB/CAR/PDMS SPME fiber (Supelco, Bellefonte, PA, USA). The samples underwent an initial incubation time of 10 min at a temperature of 40 °C with an agitator speed of 250 rpm. After that, the compounds from the headspace were extracted for 20 min under the same temperature conditions. Subsequently, the VOCs were desorbed for 3 min at the injector port, which was set at 250 °C prior to the GC-GC analyses. The GC-GC data acquisition was conducted on a Pegasus BT 4D GC**×**GC-TOFMS instrument equipped with a cryogenic modulator (LECO Corp., St Joseph, MI, USA). A mid-polar first-dimension column (^1^D) (Rxi-624Sil MS, 30 m × 0.25 mm id × 1.4 μm df; Restek Corp., Bellefonte, PA, USA) was combined with a highly polar second dimension (^2^D) column (Stabilwax, 2 m × 0.25 mm id × 0.5 μm df; Restek Corp., Bellefonte, PA, USA) for all chromatographic separations. The primary GC oven ramped from 40 to 220 °C with a 5 °C/min gradient, resulting in a total run time of 36 min. The secondary oven was regulated with a temperature offset of + 20 °C relative to the primary oven. The transfer line was maintained at an isothermal temperature of 250 °C. A modulation period of 2.5 s was used for all separations, consisting of a 0.75-s hot jet and a 0.5-s cold jet. Mass spectra were acquired at a speed of 200 spectra/second between mass channels (m/z) 33 and 450. All measurements were performed in triplicate.

### Data Analysis (GC×GC-TOFMS)

The acquired data were processed using ChromaTOF Tile software (ver. 1.2.6.0, LECO Corp, RRID:SCR_023077), with a ^1^D × ^2^D tile size of 5 modulations (12.5 s) × 15 spectra (75 ms). The software generated a peak table containing 398 variables for the 45 chromatographic measurements. Analyte identification was conducted by comparing the obtained spectra against the NIST17 library (RRID:SCR_006452). Analytes with similarity values greater than 800 were tentatively identified, while analytes with similarity values below 800 were considered unknowns (Unk). Data from the analysis are provided in Supplementary 3. Prior to chemometric analysis, missing values, which accounted for 2.4% of the data, were imputed using the column median. The data were then scaled using the z-score method, which involved centring each column to a mean of 0 and scaling it by the standard deviation. Finally, the data were normalized by probabilistic quotient normalization (PQN) [41]. Statistical analysis and data visualization were performed using in-house MATLAB v. R2019b scripts (MathWorks, Natick, MA, USA, RRID: SCR_001622). Feature selection was performed using the machine-learning algorithm Random forest [55].

### Phylogenetic Analysis

Phylogenetic analysis was conducted using three loci (ITS, *tubb, and tef1-α*) and the strains that were used for GC–MS analysis. The strains can be found in Table 1, along with the accession numbers for DNA sequences. DNA was extracted from strains cultivated on Sabouraud broth (Sigma-Aldrich, St. Louis, USA) using the Quick-DNA Fungal/Bacterial miniprep Kit (Zymo Research, Irvine, USA). MyTaq polymerase (Meridian Bioscience, Wilford, USA) was used for the PCR. The primers for the three genetic loci were chosen according to Čmoková et al. [56]. The PCR products were sequenced using the Sanger method on ABI Prism 3130XL (Applied Biosystems, Waltham, USA). The analysis was supplemented with data from reference type strains available in the NCBI database (https://www.ncbi.nlm.nih.gov/). The sequences were aligned using the FFT-NS-i option implemented in the MAFFT online service [57]. The alignments were trimmed, concatenated, and analysed using Maximum likelihood (ML) and Bayesian inference (BI) methods. The best partition scheme and substitution models were determined through computations. Partitioning schemes and substitution models were selected using PartitionFinder 2 based on the Bayesian information criterion [58]. Introns, exons and segments of the ITS region were treated as independent datasets. The optimal partitioning schemes of the analysed sequences can be found in Supplementary 1. The ML analysis was performed using IQ-TREE version 2.1. 2 [59]. Nodal support was determined using nonparametric bootstrapping (BS) with 1000 replicates. The phylogenetic tree’s graphical outputs were generated using iTOL (Interactive Tree of Life) [60].

## Results

### VOCs Spectra Comparison

**Fig. 1:**
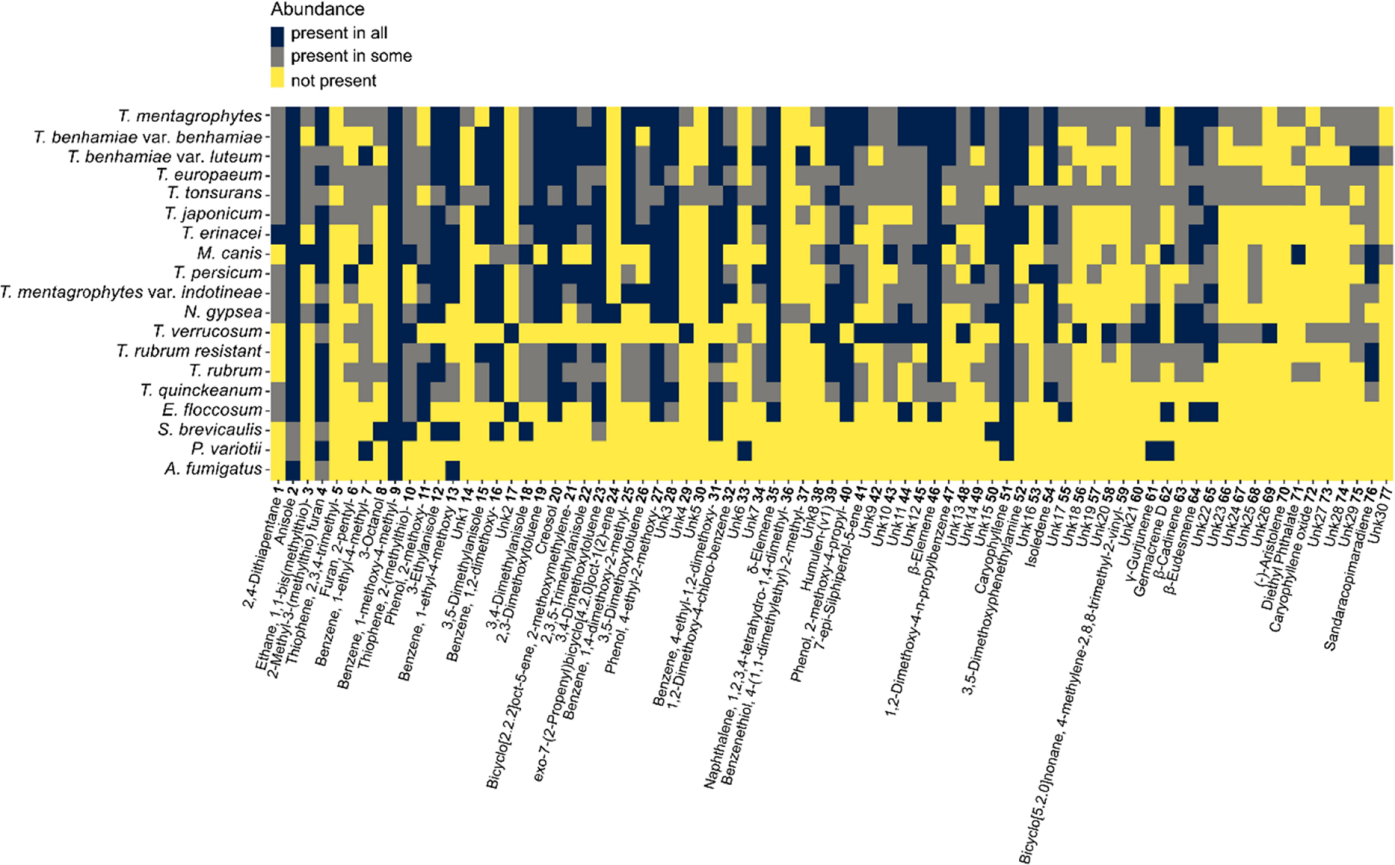
Overview of the features detected by gas chromatography-mass spectrometry (GC–MS) in all the dermatophyte and non-dermatophyte species, based on the presence/absence of a feature across the strains of the particular species

The GC–MS approach was chosen for the comprehensive analysis of 40 strains from 15 dermatophyte taxa (Table 1, Supplementary 2). To compare the overall production of volatiles among species, we selected 77 features (predicted volatile compounds) that represent main peaks in the spectra (Fig. 1). Three non-dermatophyte fungal pathogens (*A. fumigatus*, *P. variotii* and *S. brevicaulis*) were chosen for comparison.

**Fig. 2:**
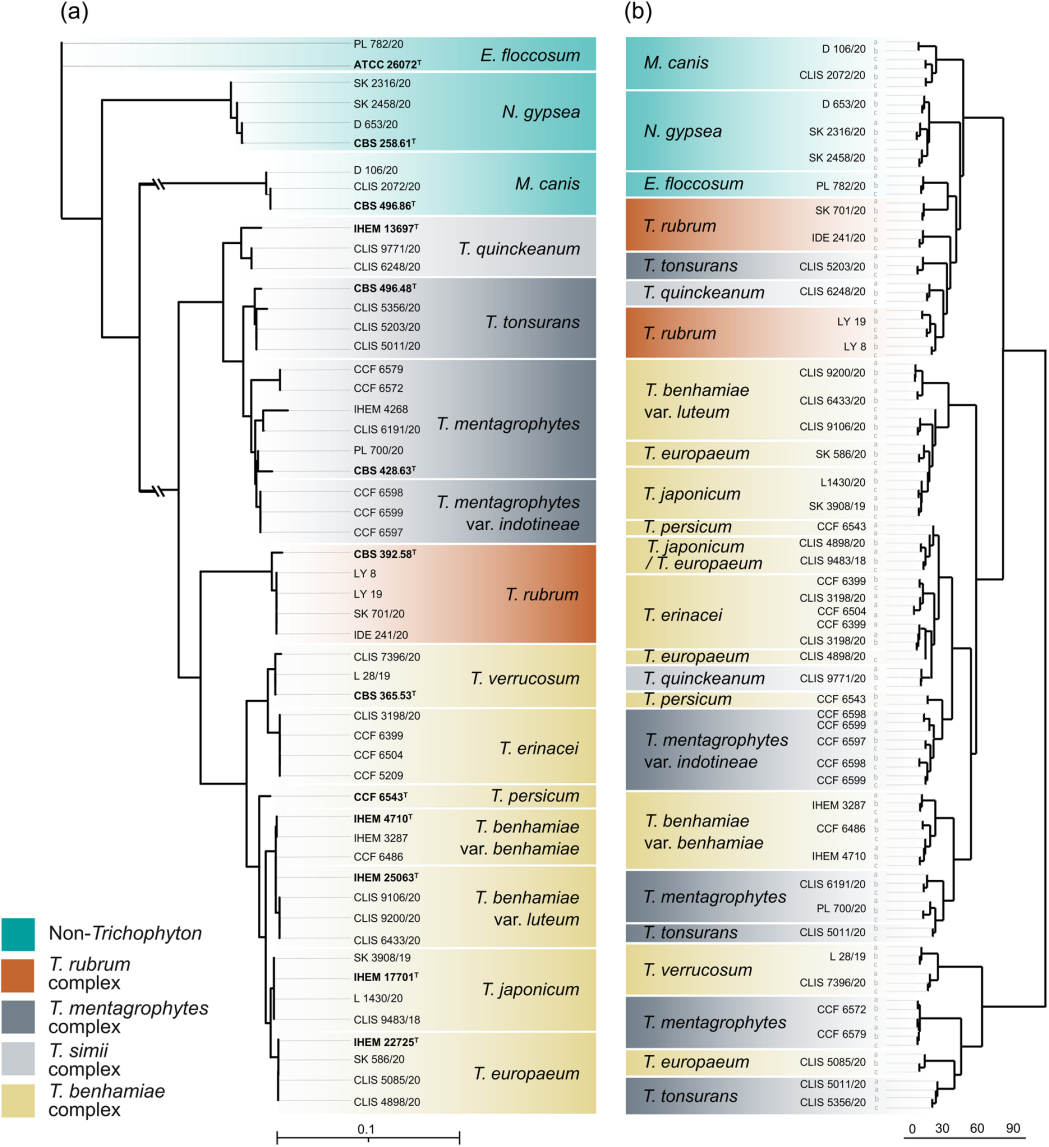
Comparison of dendrograms based on sequence markers (**a**) and VOCs spectra (**b**) of dermatophytes. Maximum likelihood multilocus (ITS, *tubb*, and *tef1-α*) tree (**a**). The ex-type strains are designated with a superscripted T. Hierarchical clustering analysis on VOCs spectra analysed by gas chromatography-mass spectrometry (GC–MS) (**b**). Three biological replicates per each strain were studied

The three biological replicates predominantly clustered together, except for *T. tonsurans* and *T. persicum*. The major clades defined by multigene phylogeny are for the most part not respected by those obtained based on volatile spectra. However, strains of the same species, as defined by multigene phylogeny, mostly clustered together in the dendrogram created based on volatilome spectra (Fig. 2). Some phylogenetically related species also tended to cluster together based on VOCs spectra, such as some members of the *T. benhamiae* complex including *T. benhamiae*, *T. europaeum*, *T. persicum*, *T. japonicum* and *T. erinacei*. However, the clade of the *T. benhamiae* complex did not include *T. verrucosum* and one isolate of *T. europaeum* (CLIS 5085). Additionally, species within the *T. benhamiae* complex mixed with some isolates of *T. mentagrophytes*, *T. tonsurans* and *T. quinckeanum* (Fig. 2). The species *T. mentagrophytes*, *T. quinckeanum*, *T. tonsurans* and *T. europaeum* showed high intraspecies variability in volatile spectra and different isolates of these species did not cluster together (Fig. 2). The Mantel test was conducted to assess the relationship between the phylogenetic distances and the distances calculated from GC–MS data. The analysis yielded a Mantel statistic r = 0.4185 (*p*-value = 0.004) indicating a moderate positive correlation. Additionally, our observed statistic exceeded the 99% quantile of the permutation distribution, further reinforcing the robustness of our findings. These results imply that the variation in GC–MS data may reflect underlying phylogenetic relationships among the samples, highlighting the potential ecological and evolutionary connections captured by this analytical method.

### *Pan*-Fungal and *Pan*-Dermatophyte VOCs

Among the selected 77 features most of these features were ethers and terpenoids (Supplementary 2). Three non-dermatophyte fungal pathogens (*A. fumigatus*, *P. variotii* and *S. brevicaulis*) were chosen for comparison. Compounds 2 (Anisole), 4 (2-Methyl-3-(methylthio) furan), and 9 (Benzene, 1-methoxy-4-methyl-) appear to be common metabolites in studied dataset, as they are shared by all studied dermatophytes and non-dermatophyte species. These compounds could potentially serve as pan-fungal markers (Fig. 1). Additionally, dermatophytes share one more compound with *A. fumigatus*, five with *P. variotii*, and ten with *S. brevicaulis*. Four of these compounds, 16 (Benzene, 1,2-dimethoxy-), 23 (3,4-Dimethoxytoluene), 31 (Benzene, 4-ethyl-1,2-dimethoxy), and 51 (Caryophyllene) are present in most dermatophytes (Fig. 1). Furthermore, four compounds, 20 (Creosol), 27 (Phenol, 4-ethyl-2-methoxy-), 35 (δ-Elemene) and 46 (β-Elemene), and possibly also 40 (Phenol, 2-methoxy-4-propyl-), are absent in the volatilome of non-dermatophyte species, but present in almost all dermatophytes. *T. verrucosum* is a poor producer of VOCs and does not produce two of the compounds (20 and 27) shared by all other dermatophytes. Therefore, only two compounds (35 and 46) have the potential to serve as pan-dermatophyte markers (Fig. 1).

### VOCs of Non-*Trichophyton* Dermatophytes

All three non-*Trichophyton* dermatophytes, belonging to the genus *Nannizzia*, *Microsporum*, and *Epidermophyton*, shared most of the features with *Trichophyton* species. However, they could be distinguished from them by their relatively low production of VOCs (Fig. 1). The species *Nannizzia gypsea* produced one potentially specific VOC that was not found in other species (number 24 which is putative exo-7-(2-Propenyl)bicyclo[4.2.0]oct-1(2)-ene) (Fig. 1).

### *T. benhamiae* Complex

**Fig. 3:**
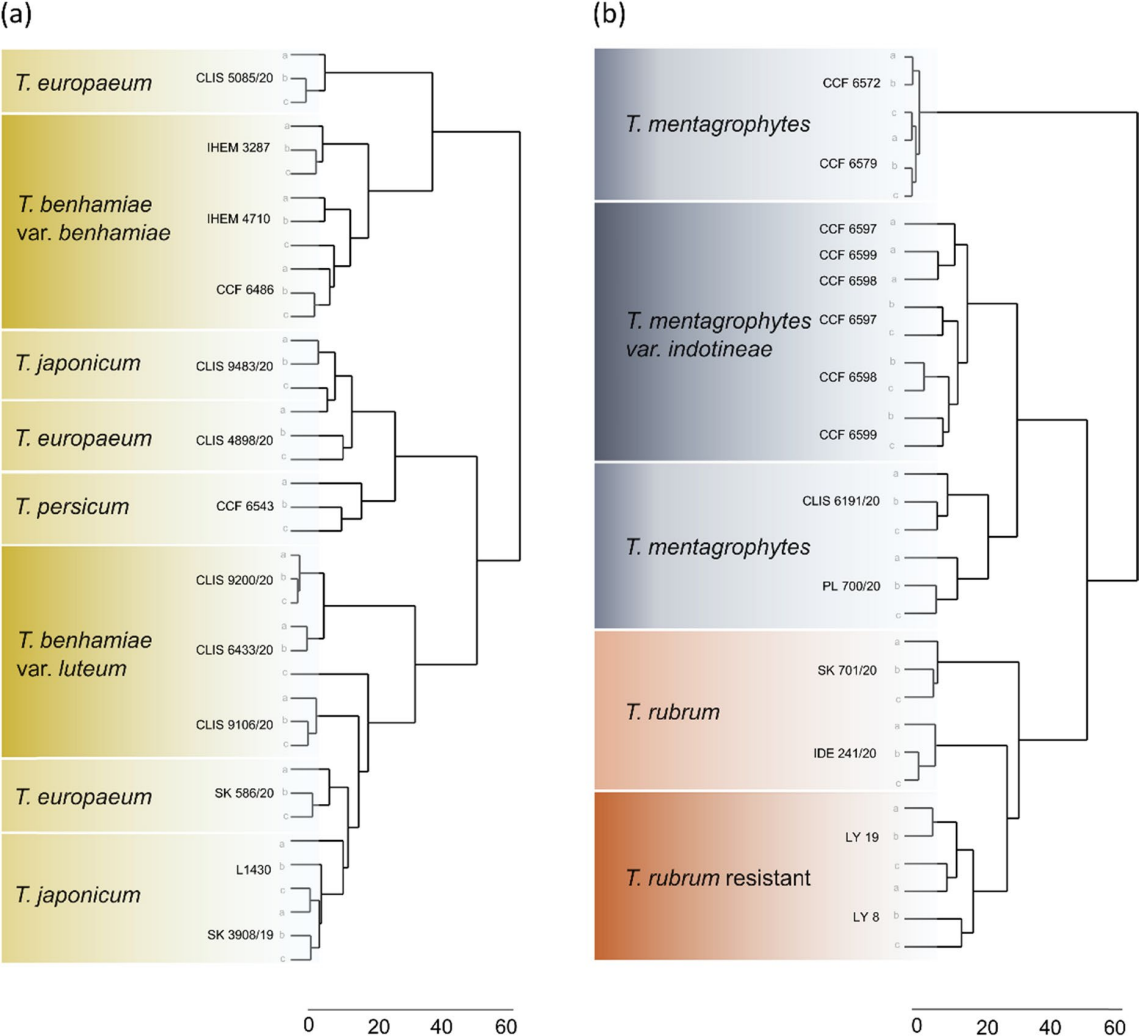
Demonstration of separation of emerging dermatophyte populations (marked with a darker colour) based on VOCs production. It showcases the identification of epidemic strains of *T. benhamiae var. luteum* within other taxa in the *T. benhamiae* clade (**a**), and drug-resistant isolates of *T. mentagrophytes* var. *indotineae* and *T. rubrum* within strains of their respective species (**b**). Hierarchical clustering analysis of strains measured by gas chromatography-mass spectrometry (GC–MS). Three biological replicates (**a**–**c**) per each strain were studied

The complex consists of eleven species and two varieties [56, 61, 62]. The most unique spectrum of VOCs was found in *T.* *verrucosum* which is clearly distinct from other members of the complex and also from other studied dermatophytes. The strains of *T. verrucosum* formed clusters within their respective species, while the strains of *T. europaeum*, and *T. erinacei* were distributed into multiple clusters. The strains belonging to *T. benhamiae* clustered according to their respective varieties (Figs. 2, 3a). The close phylogenetic relationship between *T. benhamiae*, *T. europaeum*, and *T. japonicum* was also reflected in their VOCs production and the presence of a specific feature number 5 (Thiophene, 2,3,4-trimethyl-). However, the two varieties of *T. benhamiae*, *T. benhamiae* var. *luteum* and *T. benhamiae* var. *benhamiae* can be distinguished based on VOCs production, even though they cannot be distinguished by standard phylogenetic markers, including the ITS region targeted by most diagnostic tools (Figs. 1, 2, 3a). In our analysis, *T. europaeum* and *T. japonicum* could not be distinguished by VOCs production. The most recently described species, *T.* *persicum,* could be differentiated from other *T. benhamiae* complex species based on the volatome data and clustered with *T. mentagrophytes* var. *indotineae* (Figs. 2, 3a).

### *T. rubrum* and *T. mentagrophytes* Complexes

Variability in VOCs production was observed among distinct populations of *T. mentagrophytes, T. rubrum* and *T*. *tonsurans* (Figs. 2, 3b). While *T. mentagrophytes* strains also showed high variability in sequence markers, no sequence variability was detected in *T. rubrum* and *T. tonsurans* (Fig. 2a). In the hierarchical clustering analysis based on VOCs spectra, both *T. mentagrophytes* and *T. rubrum* strains formed three clusters with distinct VOCs spectra (Figs. 2b, 3b). One *T. mentagrophytes* cluster corresponds to the variety *indotineae* as described by Švarcová et al. [63]. Two other clusters represent lineages that differ based on the age of the isolates. The more recently isolated samples (from 2020) form a sister cluster to the “*indotineae* cluster”. Older isolates (from 2017) exhibited a lower diversity in their metabolite spectra. In *T. rubrum*, one cluster consists entirely of terbinafine-resistant strains isolated in Denmark before 2016, while the other clade includes strains isolated in 2020 and originating from Czechia.

### In Depth Volatilome Analysis

**Fig. 4:**
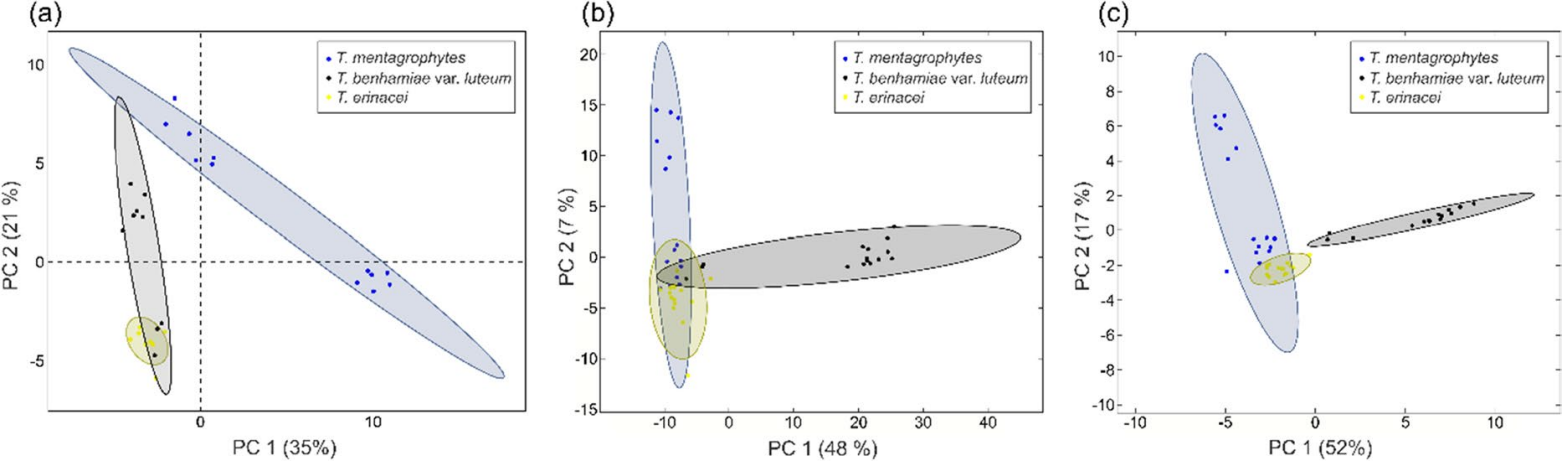
Comparison of the two approaches used in the study by comparing principal component analysis of the data measured on strains of *T. benhamiae* var. *luteum*, *T. mentagrophytes*, and *T. erinacei* by gas chromatography-mass spectrometry (GC–MS) (**a**), and by multidimensional gas chromatography with time-of-flight mass spectrometry (GC×GC–TOFMS) before (**b**), and after the feature selection (**c**)

**Fig. 5:**
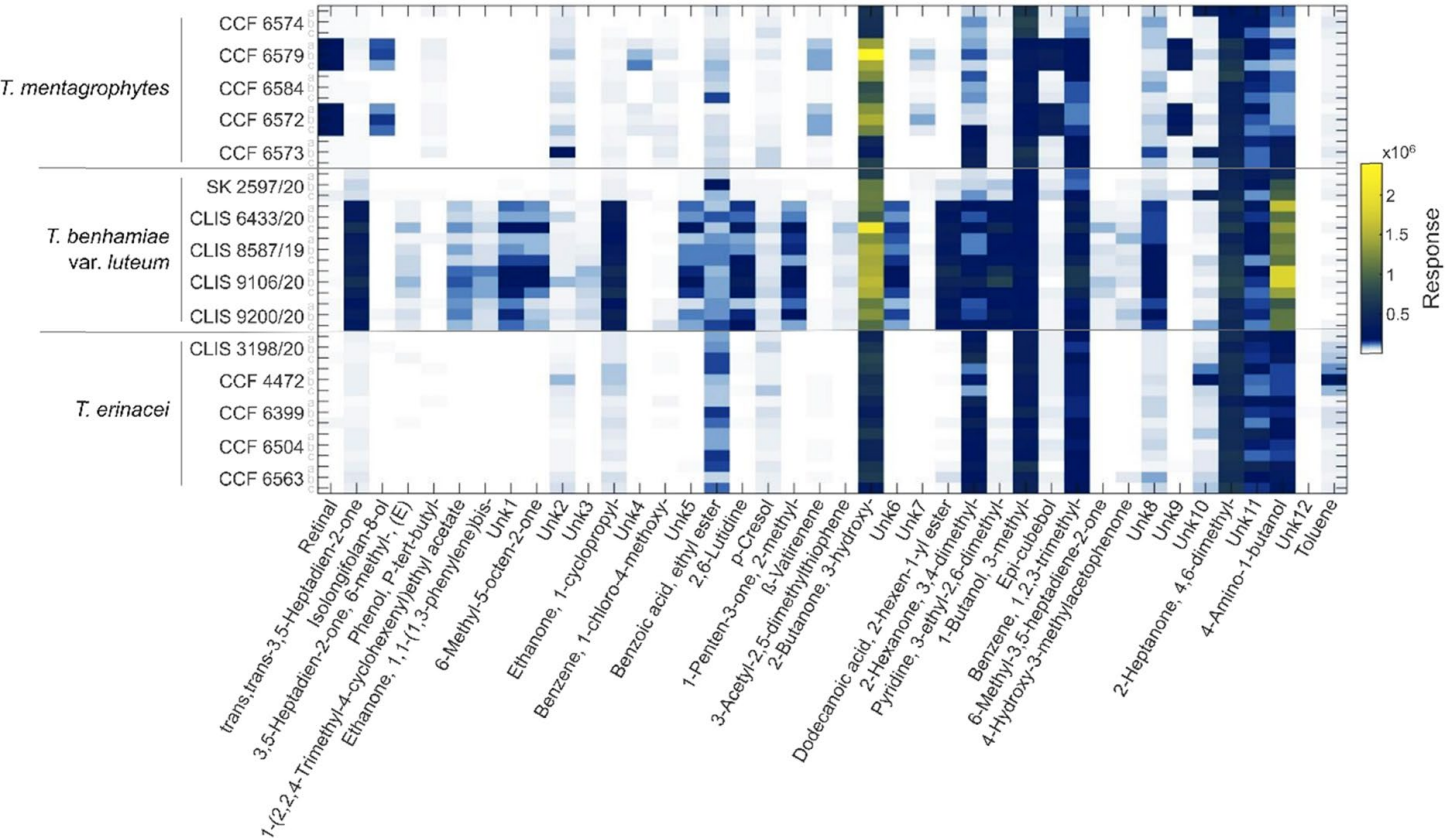
Overview of the top 40 features detected from solid-phase microextraction (SPME) followed by multidimensional gas chromatography with time-of-flight mass spectrometry (GC×GC-TOFMS) analysis of the strains of *T. benhamiae* var. *luteum*, *T. mentagrophytes*, and *T. erinacei*

For a more detailed analysis, we performed GC×GC-TOFMS analyses on five strains of each of the following taxa: *T. mentagrophytes, T. erinacei* and *T. benhamiae* var. *luteum* (Table 1). The machine learning—Random Forest analysis was applied to identify candidate chemical features that best separate the three selected taxa. All taxa showed overlap in the volatome spectra. However, we successfully separated *T. benhamiae* var. *luteum* strains from the other species after a basic feature selection process (Fig. 4). Most of the compounds discovered in these species belong to the terpenoid class. These substances were most enriched in strains of *T. mentagrophytes* through the production of sesquiterpenoids. In contrast, terpenoids were mostly depleted in the headspace of the remaining taxa. The other most well-represented class of compounds were ketones, mainly produced by *T. benhamiae* var. *luteum*. All three taxa produced compounds with sulphur, nitrogen, and chlorine (Fig. 5).

## Discussion

In this study, we explored the spectra of VOCs produced by dermatophytes and their specificity for individual taxa. The purpose of the study was to evaluate whether it is reasonable to continue developing a non-invasive diagnostic tool for dermatophytosis and species identification based on VOC spectra, as such a tool is currently missing on the market. Based on our results, it appears that identification may be possible due to the presence of some pan-dermatophyte and even species-specific VOCs. However, it remains to be determined whether these VOCs can also be detected directly from the tissue. Although the road to developing such a tool may be long, the cheaper and faster identification it could provide has the potential to revolutionize the diagnosis of dermatophytosis (Table 2).

**Table 2:** Overview of used and hypothesized methods for diagnosis of dermatophytes in clinical practice. See [5, 64–66] for further details

Analysis target	Method	Sample processing	Equipment	Approximate time	Currently used
Genotype-sequencing	PCR	Cultivation, DNA isolation	Laboratory	7–21 days	+ +
Genotype-specific	PCR, Real-time PCR	DNA isolation	Laboratory	4 h to 1 day	+ +
Phenotype-morphology	Microscopy	Cultivation	Laboratory	7–21 days	+ + +
Phenotype-proteins	MALDI-TOF	Cultivation	Laboratory	7–21 days	+
Phenotype-proteins	Lateral flow-based techniques	–	Doctor’s office	10 min	− (Possible in theory with the right equipment)
Phenotype-VOCs	GC–MS/GCxGC-MS	Cultivation	Laboratory	7–30 days	− (Possible in theory with the right equipment)
Phenotype-VOCs	Electronic nose	–	Doctor’s office	10 min	− (Possible in theory with the right equipment)

We found that a species or even population-specific spectrum of VOCs is common in dermatophytes. However, from a clinical perspective, it would be sufficient to detect any fungal or dermatophyte infection without differentiating between individual pathogens. We have identified some pan-dermatophyte and possible pan-fungal VOCs that could enable such detection. Further research on non-dermatophyte fungi and reference mixture skin microbiome communities is necessary to confirm the applicability of these markers. Previous studies have shown that VOC-based identification can achieve high sensitivity and resolution, even at the level of individual strains [28–30, 67]. Similarly, in our study, VOCs were specific to individual strains in most cases. One positive aspect of this high sensitivity is that it allows for the identification of many VOCs that can be used for specific taxa identification. Additionally, high sensitivity enables the identification of clonal populations of great clinical importance, such as strains of a clonal emerging population of *T. benhamiae* var. *luteum* with an epidemic occurrence in Europe which were clearly distinguishable from the American population of *T. benhamiae* var. *benhamiae*. These varieties cannot be differentiated using common DNA sequence markers, and their differentiation is currently only possible using microsatellite markers [56]. Similarly, the recently discussed taxon *T. mentagrophytes* var. *indotineae* significantly differs in VOCs spectra from the rest of the *T. mentagrophytes* strains in our data. Identification of this variety is not feasible using morphological identification and therefore requires molecular identification [68] or MALDI-TOF spectra analysis [69].

In our study, we found very little variability between biological replications indicating the robustness of the method. Although volatile spectra stably distinguish taxonomic units at the species level and below, their ability to capture their natural phylogenetic relationships is limited. However, in our study, we observed a moderate correlation between phylogenetic data and the cladogram derived from volatile spectra. This partial correspondence is consistent with previous findings in fungi [70, 71]. where phenotypic volatile markers are known not to be selectively neutral markers. The high VOCs intraspecific variability can be problematic in strains of certain species that share part of their VOCs spectra with unrelated taxa. Such high intraspecific variability is particularly noticeable in species that are genetically more diverse, such as *T. mentagrophytes*, while less genetically variable species tend to have more uniform production. Additionally, instability in VOCs production can be attributed to other factors. It is well-known that the fungal volatilome varies based on physiological state and environmental factors, such as substrate, incubation time, nutrients, and temperature [72–74]. The strains used in our experiments were preserved in culture collections for varying lengths of time (Table 1). Despite the different histories of individual strains, the VOC spectra of strains clustered together within certain species, suggesting a high stability of VOC production.

To determine the optimal method for sample preparation, data generation, and evaluation, we employed two approaches. Both approaches used Czapek-Dox medium and sheep wool to better distinguish between media-introduced background and compounds produced by the isolates. It is important to note that in future applications, volatile fingerprints can likely be detected even in high-background conditions and at much lower concentrations using less sophisticated instruments, such as electronic noses. However, these conditions require further testing. The GC×GC approach offers enhanced resolution compared to the GC method. Specifically, the GC×GC method identified 398 features after quality filtering, whereas GC method identified only 77 (Supplementary 2, 3). The improved resolution of the GC×GC approach allowed us to use more complex and nutritious cultivation medium, significantly reducing cultivation time. Conversely, the GC–MS method required the use of a cultivation medium (sheep wool with agar) that, under our measuring conditions, had almost no background, thus introducing fewer artifacts. However, the limited nutritional sources restricted the growth of dermatophytes. Nonetheless, it may better simulate environmental conditions during infection. We selected unprocessed sheep wool as a substrate not only for its keratin content but also because it includes other natural components such as lanolin, proteinaceous contaminant layer, and sweat residues (suint peptides) [75, 76]. These materials simulate a more complex, natural environment for dermatophytes, which can influence their growth and behavior during infection. Although sheep are not natural hosts for all species tested, several dermatophytes are known to infect sheep, such as *T. verrucosum*, which thrives on wool. Additionally, other species like *T. mentagrophytes* and *M. canis* have also been documented in cases of sheep dermatophytosis [77, 78]. Using wool from a single sheep also allowed us to maintain uniform substrate conditions across all replicates, ensuring experimental consistency. The only drawback of the GC×GC approach compared to GC is that it requires a more time-consuming data processing workflow. Nevertheless, regardless of the cultivation and analysis techniques used, each analysis provided comparable results, as they clearly distinguished certain species of dermatophytes being studied. It also shows that the differences in VOCs spectra between taxa are very robust.

In conclusion, this study involved the analysis of volatiles produced by a wide range of dermatophytes. The data obtained, along with the cultivation approach used in semi-natural conditions, makes this study the first of its kind in the field of dermatophytes. The in-depth analysis of the volatile spectrum of zoophilic species *T. erinacei*, *T. mentagrophytes*, and *T. benhamiae* var. *luteum* laid the foundation for animal model testing. Therefore, we present baseline data that could be useful for future applications, such as electronic noses.

## Supplementary Information

Below is the link to the electronic supplementary material.

Supplementary file1

Supplementary file2

Supplementary file3

Supplementary file4

## Data Availability

The conclusions drawn from this study are substantiated by data that is readily available to the public. Supplementary 2, 3 and 4 contain raw and normalized data from the GC–MS and GC×GC-TOFMS analyses. The DNA sequences of the three loci (ITS, *tubb*, and *tef1-α*) derived from the dermatophyte strains utilized in this study have been deposited to the GenBank database (refer to Table 1 for more details). Furthermore, Supplementary 1 provides comprehensive information about the partitioning, the most suitable substitution models, and the characteristics of the alignment.
